# Targeting the Tumour: Cell Penetrating Peptides for Molecular Imaging and Radiotherapy

**DOI:** 10.3390/ph3030600

**Published:** 2010-03-11

**Authors:** Veerle Kersemans, Bart Cornelissen

**Affiliations:** Gray Institute for Radiation Oncology and Biology, University of Oxford/Old Road Campus Research Building, Off Roosevelt Drive, Churchill Hospital, Oxford OX3 7DQ, UK; Email: Bart.Cornelissen@rob.ox.ac.uk (B.C.)

**Keywords:** cell penetrating peptides, molecular imaging, radioimmunotherapy

## Abstract

Over the last couple of years, the number of original papers and reviews discussing various applications of cell penetrating peptides (CPPs) has grown exponentially. This is not remarkable since CPPs are capable of transporting the most varying cargo across cell membranes which is one of the biggest problems in drug delivery and targeted therapy. In this review, we focus on the use of CPPs and related peptides for delivery of imaging contrast agents and radionuclides to cells and tissues with the ultimate goal of *in vivo* molecular imaging and molecular radiotherapy of intracellular and even intranuclear targets.

## 1. Introduction

Since their discovery in 1988, cell penetrating peptides (CPPs) have been extensively studied and in 2009 alone, over 200 papers were published, including 10 reviews [1,2,3,4,5,6,7,8]. New CPPs are being reported almost monthly [9,10,11,12,13,14,15]. This demonstrates that CPPs have become a very popular tool for targeting various cargos intracellularly, thus bypassing the very permeable-selective nature of the plasma membrane. The success of CPPs lies in their potential to unlock intracellular (and even intranuclear) targets for drug delivery and molecular imaging, by the adaption of a wide variety of agents ranging from peptides to antibodies and (drug-loaded) nanoparticles. This can provide a new approach to molecular imaging and therapy as many targets aren’t located on the cell surface but intracellular or even intranuclear.

With a publication in Science, Schwarze *et al*. demonstrated the *in vivo* potential of CPPs: intraperitoneal injection of beta-galactosidase protein, fused to the TAT protein, resulted in delivery of the biologically active fusion protein to all tissues in mice, including the brain [16]. Since then, it was clear that these results could eventually open new possibilities for direct delivery of proteins into patients in the context of protein therapy. However, despite their tremendous potential, the majority of CPP research to date is limited to elucidating their cell uptake mechanisms and to the exploration of novel compounds or intracellular targets in cell-based systems. Although CPPs don’t seem to be toxic to cells and organisms, only a few papers report on the use of CPPs for *in vivo* applications such as delivery of peptides and proteins to target different diseases including cancer, asthma, apoptosis, ischemia, stimulating cytotoxic immunity and diabetes [17,18,19,20,21]. As Chen *et al*. suggest, this might be a reflection of the uncertain technical challenges and costs of introducing an extra element into the structure of the compound and subsequent evaluation of its properties [22]. In contrast to the variety of papers that describe the delivery of oligopeptide/protein and nucleic acids or analogs, this review will focus on the use of CPPs in molecular imaging with applications diverse from optical, magnetic resonance and nuclear medicine imaging.

## 2. Uptake Mechanisms and Repercussions for Applications

CPPs are small polypeptides that contain several positively charged amino acids such as lysine or arginine, or have sequences that contain a pattern of alternating polar and non-polar, hydrophobic amino acids. These two types of structures are referred to as polycationic or amphipathic, respectively. Despite the numerous CPP publications, the mode of cell membrane translocation of most CPPs remains controversial as no single, unified uptake mechanism has been isolated. As indicated by Heitz *et al*. [8], this can be partly explained by the fact that different labs use different methods which are often not comparable. Moreover, many studies rely on the use of fluorescein dyes which can give rise to misleading data as the molecule is pH sensitive and its effect on the cellular dynamics cannot be overlooked [23]. Also, extensive peptide degradation once the molecules reach the intracellular compartment can hamper cargo delivery.

Although no general uptake mechanism has been elucidated yet, it is now believed that proteoglycans, through electrostatic interactions, play an important role in the initial contact between CPPs and the cell surface [24,25]. Then, following actin remodelling, CPP-mediated transport can occur through different endocytosis routes [26]: *via* caveolae [27], macropinocytosis [28,29] through a clathrin-dependent pathway [30], *via* a cholesterol-dependent clathrin-mediated pathway [31] or in the trans-Golgi network [32]. Additionally, it is important to emphasize that the cellular uptake pathway is driven by several parameters, including the nature of the CPP, its ability to interact with the cell membrane and membrane lipid components, the nature, type and active concentration of cargo, the linkage CPP-cargo, the ratio CPP:cargo, the cell type, the membrane composition and a possible fluorophore or other reporter group such as a metal chelator [8,24,33,34]. A recent review distinguishing the many forms of clathrin-independent endocytosis even suggested that a single cell may have several different mechanisms for internalisation available to itself, and that for each one there is a requirement for unique and universal proteins and lipid components [34]. Although one might reason that ‘the mechanism of uptake isn't important as long as the application works’, it is still indispensible to understand the uptake mechanism in order to optimise CPP mediated targeting.

In contrast to the many papers about cellular uptake mechanisms, little is known about their intracellular trafficking which is equally as important to allow the cargo to reach its target within the cell. Since endocytosis is likely to be involved, one can expect lysosomal degradation of the CPP and its cargo. Thus, as a result, endosomal escape remains a bottleneck in CCP-mediated drug/cargo delivery as only a few CPPs contain an endosomal breaker property. Although this problem might be overcome by the addition of an endosomal escape sequence, little research has been conducted. A recent review [6] has presented some solutions to this specific issue. 

## 3. Applications of CPPs for *in Vivo* Molecular Imaging (in Oncology)

In a review previously published elsewhere, we have given an overview of the state of the art in the field of molecular imaging and molecular radiotherapy using CPPs [3]. Here, we will highlight some of the applications of CPPs in molecular imaging and molecular radiotherapy, and give a synopsis of some of the most interesting work that has been reported in the literature since then by us and others.

**Figure 1:**
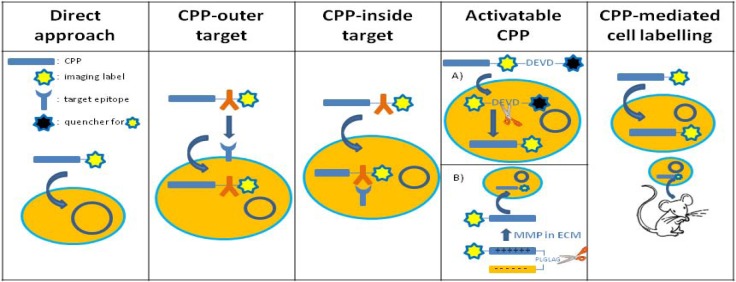
A schematic overview of the various CPP-construct approaches for molecular imaging.

The use of cell penetrating peptides in molecular imaging can be organised by different categories: (i) direct application, (ii) CPP constructs with an intracellular or intranuclear target, (iii) CPP constructs with an extracellular target, (iv) activatable CPPs, and (v) use of CPP to track prelabelled cells. Although the latter is technically not a molecular imaging technique, we have included its use in this review. For a schematic overview, see Figure 1.

### 3.1. The Direct Approach

CPP constructs with the general structure (CPP-label) have been used in the past to study the behaviour of (radio-)labelled CPPs *in vivo*. Polyakov *et al*. were one of the first groups to describe the biodistribution of a labelled CPP, with the final goal of developing novel applications in medical imaging and radiotherapy [35]. These authors reported a TAT-peptide (GRKKRRQRRR) modified with KGC motif, to provide a N(3)S core for labelling with ^99m^Tc (and possibly ^188^Re). ^99m^Tc-TATp was rapidly taken up into Jurkat cells (t_1/2_ < 2 min), and washout of ^99m^Tc was also very quick. Nuclear (possibly nucleolar) uptake of a FITC-labelled version of the same TATp was also demonstrated. Biodistribution studies of ^99m^Tc-TATp showed extremely fast renal clearance. The authors mention the possibility of further derivatization for targeted applications for imaging and therapy.

A similar approach was reported two years later by Bullok *et al*. [36], who synthesized a FITC-labelled TATp (RKKRRQRRRGC), as well as a DTPA-labelled or N-terminal histidine variant, for radiolabelling with ^99m^Tc(CO)_3_ or ^188^Re(CO)_3_. As reported by Polyakov, rapid renal clearance was also observed, but with marked differences in liver and kidney uptake for radiolabelled DTPA- or histidine- TATp, compared to the study by Polyakov. No brain uptake was observed by either Polyakov or Bullok.

An Alexa Fluor 595-conjugated TAT-peptide was used by the Barnett group [37]. The complex was topically applied to the rat retinas, and found to specifically internalize into retinal ganglions. Even though the addition of different cargo to the same CPP might drastically change its biodistribution, the group went on to develop a cell-specific ocular drug delivery system using the TAT peptide [38], as well as a caspase-activatable CPP-complex for molecular imaging of apoptosis (see below) [39,40].

Chen *et al*. describe the conjugation of CPPs to quantum dots (QD) [41]. QDs have higher extinction coefficients, higher quantum efficiency and narrower excitation and emission spectra compared to the ubiquitously used fluorophore like the Alexa Fluor family dyes. The authors show that TAT-conjugation of QDs is possible, but that their internalization pathway is markedly different compared to that of TAT-FITC conjugates.

In an analogous report, Medintz and co-workers reported the translocation across the cell membrane of QD cargos, linked to fluorescent proteins, mediated by a (His)_8_ linked to poly-arginine CPP [42]. CPPs were successfully linked to QDs either by metal affinity self-assembly, or *via* a biotin-avidin conjugation. It was shown that protein-QD-CPP complexes were readily internalized into HEK293 cells, and that QD fluorescence did not suffer from quenching, thereby offering a big advantage for repeated imaging of CPP-QD complexes. The constructs were mainly taken up by the endolysosomal compartment of cells. The authors predicted that the basic architecture of their construct could help in the intracellular tracking of proteins, and potentially drug delivery. Although QDs have obvious advantages over fluorophores, they are not used extensively for *in vivo* molecular imaging, because of their toxicity *in vivo*. The less protected the QD core material is, the faster the appearance of cellular toxicity due to Cd^2+^ or Se^2–^ ion release from the QD core. Then again, only little is known about the excretion process of polymer-protected quantum dots from living organisms. These and other questions remain to be answered before QD applications in molecular imaging can be approved for clinical use [43].

### 3.2. CPP Constructs with an Intracellular or Intranuclear Target

Most studies using a (CPP-label) construct were set up to meet intermediate goals, such as determination of the biodistribution of CPPs, feasibility, and safety studies. The obvious advantage of the use of CPPs in molecular imaging is, as mentioned above, that it unlocks the potential of intracellular and even intranuclear targets. Imaging probes with the general structure (CPP-targeting-label) have been used to image a kaleidoscope of proteins, including targets involved in apoptosis; cell cycling; DNA damage repair; and overexpression of various mRNA species. Constructs targeted against proteins make use of antibodies, antibody fragments and derivatives, or targeting peptides. CPP-constructs for DNA/RNA imaging contain modified antisense (morpholino) oligonucleotides or peptide nucleic acids (PNAS) to bind to their target. Contrast for imaging originates from the differential in cellular retention between cells that express the target (specific retention of the CPP probe) compared to cells not expressing the target. Most of the complexes described so far, including our own work, do not exhibit any form of active tumour specific translocation, and uptake in the tumour is only higher than normal tissues because of the enhanced perfusion rate (EPR) and hyperfenestration.

The first to report the use of a CPP-targeting-label construct, was the Heidelberg-based group of Eisenhut *et al*. [44]. As an alternative to the prevailing Annexin V targeting of PS to image apoptosis [44,45], a caspase 3 targeting retro-inverso TAT-peptide construct was synthesized. This used a DEVDG-motif for caspase 3 binding, because this motif is known to be cleaved by downstream caspases. They successfully radioiodinated TAT_57-49_-yDEVDG, but only showed a mere two-fold higher uptake in apoptotic cells compared to normal controls. They also found that for all other synthesized TAT-DEVDG constructs, CPP-mediated uptake was hampered by the morphological changes in the apoptotic cells.

The Reilly group have extensively studied radiolabeled TAT-antibody complexes. Hu *et al*. first reported on TAT-peptide conjugated anti-p21 antibodies [46,47,48]. Radioiodinated TAT-peptides were site-specifically conjugated to the Fc tail of the IgG. These radioimmunoconstructs were shown to internalize into breast cancer cells, and translocated to the nucleus (since the TAT-peptide sequence includes a nuclear localisation sequence (NLS)), where it could bind to p21, a cyclin-dependent kinase inhibitor and a regulator of the cell cycle. In cells exposed to Endothelial Growth Factor (EGF), p21 was upregulated, and ^123^I-antip21-TAT retention was increased by 50%. *In vivo*, however, the direct radioiodination of the construct resulted in low stability [47]. The construct was rapidly deiodinated and lead to a relatively low upregulation of retention (1.7-fold) in xenograft tumours that overexpressed p21. Therefore, in a later paper, the group changed their radiolabeling approach, and used ^111^In with DTPA as a metal chelator, to produce a more stable radionuclide bond and the target was switched from the EGF-induced p21 to the trastuzumab-induced p27 protein [49]. The expression of p27, a cyclin-dependent kinase inhibitor exerting similar functions compared to p21, is upregulated after exposure of cells to the HER2-receptor blocking antibody trastuzumab (Herceptin). Synthesis, cell penetration, nuclear localization and p27 interaction were all demonstrated. Retention of ^111^In-anti-p27-TAT was increased 16-fold in p27-induced cells after exposure to trastuzumab, compared to untreated cells. However, *in vivo* uptake of ^111^In-anti-p27-TAT in tumour xenografts was rather low (4.8 ± 0.1 %ID/g) and this was only mildly increased (about 30%) in tumours of animals treated with trastuzumab, possibly because of the low basal expression levels of p27. Even in the case of an upregulation, expression is still low and cannot guarantee the specific retention of the imaging probe necessary for image contrast. Consequently, when our group used the same (label-IgG-TAT) approach with the final goal of imaging DNA damage after chemo- or radiotherapy, a much more abundant target was chosen: the DNA double strand break (DSB) repair protein γH2AX. The histone H2A X-isoform is readily phosphorylated after DSB formation, forming γH2AX, which appears as foci around the DSB with thousands of copies per focus, acting as a scaffold for further DNA repair machinery [50]. Our group has shown colocalisation *in vitro* between γH2AX foci and fluorescently labelled anti-γH2AX-TAT in irradiation-damaged cells, increased retention of the probe in irradiated cells (16-fold) and *in vivo* fluorescent imaging of DNA damage following external beam irradiation (4 and 10 Gy) of a tumour xenograft [51]. Also, using ^111^In-anti-γH2AX-TAT for SPECT imaging, we were able to demonstrate 6-fold increased uptake in a tumour xenograft of the probe after DNA DSB caused by either external beam irradiation (10 Gy) or intraperitoneal administration of the chemotherapeutic agent bleomycin.

Many applications of the (CPP-targeting-label) can be found in the DNA/RNA targeting field, using varying targets, labels and conjugation techniques, which makes direct comparison between reports difficult. For DNA/RNA CPP-imaging field, no systematic, comparative study has yet been set up to determine the optimal probe design. Galazzi *et al*. were the first to describe a retro-inverso CPP (araqraaarayg) –conjugated anti-bcl2 peptide nucleic acid (PNA), conjugated to either ^111^In *via* DOTA, or tetramethylrhodamine (TMR) [52]. Using the fluorescent TMR construct, they showed high uptake of the targeted construct, but not of a scrambled control PNA sequence in cells known to overexpress bcl2, a proto-oncogene involved in apoptosis homeostasis regulation. To target Rialpha RNA, Zhang *et al*. used a morpholino oligonucleotide linked to a N2S2 ^99m^Tc binding core [53]. Heckl *et al*. produced a anti-*cMyc* PNA linked to the CPP TQVKIWFQNRRMKQKKC with a disulfide bridge, conjugated to Gd *via* DOTA, for MRI imaging, and showed increased retention of *cmyc*-specific PNA-CPP sequences compared to scrambled PNA controls [54]. In their 2007 paper, Wang *et al*. used a ^99m^Tc labelled anti-survivin RNA morpholino, synthesised using biotin-streptavidin-biotin linkeage to TAT [55]. In the same year, Su *et al*. reported their synthesis of a double labelled Gd- FITC-construct for MRI and fluorescent imaging of the TAT-peptide and a PNA against dsRed RNA, linked by a PEG linker [56]. They did not show dsRed RNA specificity, but in a recent follow-up paper, Mishra *et al*. investigating differential uptake/retention in DsRed-transfected cells and showed a slightly higher uptake of the CPP-PNA construct compared to non-sense controls or non-transfected cells. In the *ex vivo* setting, the authors demonstrated high CPP-mediated delivery of the fluorescent label into liver, bladder, kidney and splenic tissues, but no uptake in brain or blood. *In vivo* MRI imaging was not performed. Nonetheless, amongst the few papers using these DNA/RNA targeting CPP constructs, Meshra *et al*. are the only ones that investigated differential uptake/retention in cells that either express or not express the target, and together with the use of scrambled non-sense controls, proved an indispensible check on probe specificity.

### 3.3. CPP Constructs with an Extracellular Target

All the CPPs used so far in the synthesis of constructs for molecular imaging and drug delivery are inherently non-specific for tissue type. At best, they exert some specificity by entering in some cells more than others, a discrepancy that is not yet fully understood, or enhanced by EPR. To try to resolve this, some groups have started to combine the membrane transduction capabilities of CPPs with tumour targeting moieties, e.g., other peptides (called CTPs, cell targeting peptides [5]), proteins or antibodies. Indeed, the addition of a CPP to an imaging probe against an extracellular target can increase imaging contrast by inducing cellular internalisation of the probe, thereby reducing washout and increasing retention [57]. Moreover, CPPs induce a more homogeneous delivery of the imaging probe to the tumour, thus interrogating a larger part of the tumour.

The Reilly group used ^111^In-labelled trastuzumab, an anti-HER2 antibody used clinically, conjugated to the SV-40 NLS sequence [58,59] for radioimmunotherapy of HER2 overexpressing tumours. The NLS sequence induced increased nuclear uptake of Indium-111 to increase, and subsequently decreased both the clonogenic survival of HER2-overexpressing tumour cells, and tumour xenograft growth inhibition *in vivo*. Moreover, the SV-40 NLS sequence has some CPP capability as well, and increased cell uptake compared to unconjugated ^111^In-trastuzumab was observed.

Jain *et al.* used CPPs to improve the tumour-retention of a tumour targeting single-chain fragment of a monoclonal antibody [57]. They synthesized ^125^I-labeled constructs of a divalent single chain fragment (scFv)2 derived from anti–tumour-associated glycoprotein-72 monoclonal antibody CC49, linked to either TATp or penetratin. They showed that tumour uptake and the ratio of tumour-to-normal tissue at 24 hours in LS174-xenografts in mice to be increased significantly when CPPs, especially penetratin, were attached to the sc(Fv)2 fragments.

The Langel group have published two papers so far where they combine the CPP pVEC with a cyclic peptide PEGA (CPGPEGAGC), which accumulates in breast cancer vasculature [60], or a linear breast cancer homing peptide, CREKA, which binds to clotted plasma proteins and fibrin-like structures [61]. Chlorambucil, a cytotoxic agent, conjugated to pVEC-PEGA was shown to reduce clonogenic survival of MCF-7 cells. The fluorescently labelled pVEC-CREKA was taken up in MCF7 cells as well, but to a lesser degree than plain fluorescently labelled pVEC without the CREKA peptide attached. No *in vivo* data were reported. FITC-labelled pVEC-PEGA was internalized into MDA-MB-231 cells, but to a lesser extent than FITC-pVEC. FITC-pVEC-PEGA was only found in MDA-MB-435 tumour xenografts after intravenous injection in mice, whereas uptake of FITC-pVEC without PEGA was not only observed in the tumour, but also in the lungs, liver and skin. In summary, even though attachment of a cell targeting peptide to a CPP decreased the membrane transduction capability of the construct, the increased tissue selectivity reduced normal tissue uptake drastically. Non-invasive imaging in a live animal has yet to be reported, but the advantages of this approach are evident.

### 3.4. Activatable CPP Constructs

Another solution to the problem of tissue non-specificity of CPPs is the development of so-called “activatable” CPPs. Constructs have been described that are “activated” either outside or inside the cells. In both cases, a detectable signal is only generated in tissues with a specific cleaving enzyme.

A very elegant study by the group of Nobel laureate Roger Tsien on the use of a Cy5 conjugated poly-D-arginine CPP, linked to a CPP-blocking poly-D-glutamate sequence by a MMP-2, -9 and -14 cleavable PLGLAG linker was published in 2006 [62,63]. In plasma, the poly-D-glutamate moiety stops the transduction action of the CPP. When the linker is cleaved by the matrix metalloproteinases MMP2, -9 or 14, important regulatory enzymes in the extracellular matrix of many tumour types [64,65,66,67,68], the CPP-Cy5 conjugate is “released”, and can enter the surrounding tumour cells. They have also shown that the activatable form is cleared much slower from the blood than the “plain” Cy5-polyarginine CPP [61]. The contrast between MMP-expressing tumour and non-target tissue was excellent and demonstrates that this approach is distinctly different to the use of (radio-)labelled MMP-inhibitors for molecular imaging of MMPs [69,70,71,72,73], as the former measures MMP activity, and the latter, MMP expression.

Watkins applied a similar approach, using SGRIGFLRTA, an MMP-14 cleavable linker, between a CPP (octo-D-arg) and various poly-glutamate “attenuation” sequences [74]. The CPP was conjugated to a single amino acid chelate (SAAC) for conjugation to ^99m^Tc(CO)_3_. *In vitro*, they showed MMP-14 specific uptake into MDA-MB-231 breast cancer cells, which decreased significantly upon chemical MMP-14 inhibition. No *in vivo* studies have been reported so far.

Caspases are a well-known class of apoptosis-related enzymes, capable of cleaving proteins at specific DEVD sequences. This approach was used by Bullok *et al*. to engineer a construct in which the fluorescent dye Alexa Fluor 647 was coupled to the quencher QSY21 *via* DEVD [36,75]. The whole was conjugated to a CPP, which enabled the probe to be internalised into cells, where caspase activity cleaved the link between the quencher and the fluorescent dye, causing a fluorescent signal to be generated. Here, not the CPP, but the fluorescent dye is activated. An improved version of this construct was recently reported by the same group, using a modified all-D-CPP [40]. The latter was used for *ex vivo* single cell apoptosis imaging in rat retinal ganglions, and showed that TUNEL staining for apoptosis corresponded well with localisation of the activated caspase-cleaved probe [37,39]. For obvious reasons, this technique is limited to optical imaging.

In an interesting study by Yeh *et al*., nuclease-resistant molecular beacons were delivered into cells using a TAT-peptide for real-time detection of viral infection [76]. The molecular beacon consisted of a fluorophore and a quencher on opposite ends of an oligonucleotide sequence, which was specific for a non-encoding part of the viral RNA. Under normal conditions the molecular beacon is wrapped in on itself, and the quencher blocks the fluorophore from emitting light. However, when encountering the antisense viral RNA sequence, the molecular beacon linearizes, increasing the distance between the fluorophore and the quencher. Fluorescence thus only occurs in virus-infected cells.

Dmitriev *et al*. have published data on an oxygen-sensitive Pt-porphyrin structure, which shifts its phosphorescent emission lifetime from 30 to 66 μs depending on intracellular oxygen concentration [77]. The Pt-porphyrin is internalized into the cell by its conjugation to polyarginine or the TAT-peptide. This technology is advantageous to other methods used to date, which require facilitated transport with transfection agents. 

### 3.5. Use of CPP to Track Prelabelled Cells

The tracking of cells injected into a living animal has interested scientists over the years. This is especially so for stem cell therapy, where it is important to know the destination and survival of the injected cells. Although a lot of *in vitro* work has been published on this subject, only one *in vivo* application has thus been reported.

A gadolinium-labelled poly-arginine-peptide for tracking of preloaded cells with MRI was synthesised by Liu *et al*. and reported on in similar papers [78,79]. The authors used the Gd-CPP to successfully load mesenchymal stem cells with Gd-CPP and FITC-CPP *in vitro* and showed an absence of an apoptotic response after Gd-CPP loading. Similar findings were shown by the same group in HepG_2_ cells [80]. Although papers have been published with the same construct, important information such as the retention of the CPP inside the stem cells was not investigated, nor have any *in vivo* data been reported.

In an earlier paper from 2000, Bhorade *et al*. [81] had already used the TAT-peptide for a similar study. Gd, Dy, or ^111^In- labelled site-specifically conjugated to DOTA-TATp were synthesised. The time-specific uptake of ^111^In-Gd-TAT in murine lymphocytes was shown by gamma-counting. *In vitro* uptake of Gd and Dy compounds was demonstrated by means of *in vitro* MRI, T1-weighted imaging for Gd and T2*-weighted for the Dy labelled compound.

Prior to the experiments of Bhorade *et al*., Josephson from the Weissleder group had published on the use of CLIOs, dextran coated cross linked superparamagnetic iron oxide particles, which they conjugated to TAT-peptides, previously conjugated to FITC, and labelled the construct to ^111^In trough a DTPA chelator. This resulted in a triple-labelled CPP construct, available for multimodal imaging with fluorescence microscopy, MRI and SPECT [82]. Uptake in murine lymphocytes, activated human NK cells, and HeLa cells was shown by fluorescence microscopy using the FITC label, or using anti-dextran staining. *In vitro* T1-weighted MRI showed uptake of the paramagnetic iron oxide core into lymphocytes.

A PEG-coated iron oxide nanoparticles conjugated to the TAT-peptide co-labelled with TexasRed has been used by Nitin *et al*. They showed its uptake in MDPK and HDF cells [83]. Contrary to most other TAT-peptide applications, nuclear uptake has not been demonstrated.

The only *in vivo* study has been published by the Valliant group, who synthesised a single amino acid chelate (SAAC)-containing a variant of the TAT-peptide, and labelled it with ^99m^Tc for SPECT imaging, or Re for fluorescent imaging [84,85]. They were able to load neural stem cells successfully with ACGRKKRRQRRR[(^99m^Tc-(CO)3)–SAACQ]G, and track their path after bilateral implantation into the mouse brain [86]. The disadvantage with the use of ^99m^Tc is its short half-life, such that the monitoring of stem cell trafficking and eventual homing is limited to periods of up to 24 h.

## 4. Applications of CPPs for Molecular Radiotherapy (In Oncology)

The excellent membrane transduction capabilities of cell penetrating peptides has drawn the attention of the radioimmunotherapy community, who use radionuclides for therapy rather than imaging purposes. Radionuclides emitting alpha (^213^Bi, ^211^At), beta (^90^Y, ^131^I) or Auger electrons (^125^I, ^111^In) are used for this purpose. In most cases, as is true for radioimmunoimaging, radionuclide-conjugated pharmaceuticals, often antibodies, are targeted against epitopes on the outside of the tumour cell, or on the vasculature of extracellular matrix of a tumour. Examples of extracellular epitopes include the FDA-approved CD20-binding Bexxar (^131^I labelled) and Zevalin (^90^Y labelled). However, if after their binding to the extracellular epitope, therapeutic radiopharmaceuticals are internalised into the tumour cells, the advantages are twofold. Firstly, the retention of the radionuclide in the target tissue is prolonged, thereby increasing the dose delivered to the tumour. Secondly, in the case of Auger electron emitters, the radionuclides are brought in a closer proximity to the DNA of their target-cells, and become more cytotoxic. This is because the very low-energy electrons have an average range of approximately 10 nm, their internalisation into target cells is indispensible for effective DNA damaging and cell killing.

The Reilly group published a number of papers using the nuclear localisation sequence (NLS) of SV-40 large T-antigen as a means to increase nuclear uptake of ^111^In-labelled antibodies against a range of extracellular epitopes (CD33 and HER2) [58,59,87,88]. In doing so, they also increased cellular uptake, as a direct result of the mild membrane transduction capabilities of this NLS peptide. In all cases, conjugation of the peptide sequence to ^111^In-IgGs significantly increased nuclear translocation of the Auger electron emitter ^111^In, and significantly reduced clonogenic survival of target cells (CD33-expressing leukemic cells, or HER2-overexpressing MDA-MB-361 breast cancer cells). *In vivo*, the group was also able to show tumour growth inhibition by ^111^In-Anti-HER2-(NLS)_6_, but much less so by ^111^In-anti-HER2 (without NLS) [59].

The fact that the TAT cell penetrating peptide also harbours a nuclear localisation sequence makes it interesting to use as a delivery agent of Auger electron emitters for molecular radiotherapy. Our group has used ^111^In-labelled TAT-conjugated monoclonal antibodies against the DNA double strand break repair protein γH2AX previously for imaging of DNA damage after radio- or chemotherapy. The exact same construct, but labelled at higher specific activity (6 *versus* 1 MBq/μg) can be used for Auger electron therapy targeting existing DNA double strand breaks, to cause even more damage, and kill the cell. We have shown that ^111^In-anti-γH2AX-TAT is internalized into various breast cancer cell lines, and that it is retained 16-fold longer in X-Ray-irradiated (IR) cells that express the target γH2AX [89]. The clonogenic survival of MDA-MB-468 and MDA-MB-231/H2N cells was reduced significantly when exposed to the combination of 4 Gy IR and ^111^In-anti-γH2AX-TAT, compared to either treatment alone. The lack of extensive γH2AX expression in normal, non-target tissues greatly reduced the retention of ^111^In-anti-γH2AX-TAT in these tissues, and therefore the dose. We did not observe toxicity of ^111^In-anti-γH2AX-TAT in non-target tissues. This was predicted by Jain *et al*. who stated that “the dominance of CPP activity can be exploited if the payload is non-toxic to non-target cells if, e.g., the intracellular target is only expressed in target cells”. Jain *et al*. have made many more recommendations on the subject of molecular radiotherapy with CPP-constructs in a mini-review [90]. The authors stress the optimisation of CPP:IgG ratio, as this can influence antigen binding significantly [91,92] as well as pharmacokinetic behaviour [93,94]. Also the choice of CPP is very important for therapeutic applications, as it is for imaging.

## 5. Future Applications: Pitfalls and Considerations

Although CPPs have a huge potential to translocate a variety of cargos into the intracellular and even intranuclear compartment, they still lack cell specificity which remains a major challenge for future applications in molecular imaging and radiotherapy. Indeed, most cells when they come in contact with the CPP, and its cargo, will internalize it. As a consequence, the cargo will be taken up in the ‘wrong’ cells, which can increase normal cell toxicity and/or reduce imaging contrast significantly. However, there is light at the end of the tunnel as several research groups using different approaches are starting to address this problem. A few examples that could improve tissue (and thus tumour) targeting include local tissue administration of CPP-constructs [95,96,97], activatable CPPs that exploit the presence of tissue specific enzymes [38,39,40,44,64,65,66,67,68,75] or a difference in pH [98,99,100], the use of thermal or ultrasound sensitive carriers [101,102,103,104] and the development of chimeric structures which can include NLS-sequences, tumour-homing peptides and CTPs [105,106]. The latter might be the most interesting as such peptides, upon interactions with a receptor/protein/antigen that is exclusively over-expressed by these cells, exhibit high specificity and strong affinity for a given targeted cell line. In oncology, examples of this approach could include peptide ligands binding to VEGF, VCAM-I, erbB2, or integrins. Irrespective of the method used, it would still be a challenge to trigger the exposure of the CPP in the right place at the right time. 

Aside from the improvements on cell specificity, endosomal escape needs to be addressed. Many factors such as CPP-type, cell type, incubation conditions, etc. can alter the internalisation mechanism of the CPP, making it difficult to choose an appropriate combination for *in vivo* applications. Moreover, differences in endocytosis may lead to differences in translocation and, eventually, in endosomal entrapment or escape. Although it is not clear yet whether or not a fixed molar proportion of the internalized complex escapes the endosomes, the fact still remains that endosomal escape is believed to be the rate-limiting step in efficient intracellular delivery of the cargo to its target. El-Sayed and colleagues published a comprehensive review on the different systems currently in use for the enhancement of endosomal escape of cargos linked to CPPs [6]. These endosomal escape strategies include the use of membrane disruptive peptides and polymers, lysosomotropic agents, photosensitisers and fusogenic peptides such as DOPE. However, as El-Sayed *et al*. have stated, the lack of quantitative parameters for endosomal escape efficiency and different experimental conditions make it difficult to judge which system was the most successful. 

For *in vivo* applications, a couple important factors need to be addressed: toxicity, stability, and pharmacokinetic behaviour of the CPP-cargo complex. Again cell specific targeting springs to mind as it goes hand in hand with toxicity: if cell targeting can be improved, a lower dose is needed to achieve the same effect which could significantly decrease toxicity, especially when chemotherapeutics or radionuclides are linked to a CPP. Although it seems evident and straightforward to study toxicity and stability *in vivo*, to date only a few reports discuss these topics. Two studies, both using TAT as a CPP, [107,108] suggested that i.p. administration of TAT in mice exerts no toxicity. However, i.p. administration is seldom used in clinic and to date no general reports are published on *in vivo* toxicology of CPPs by clinically relevant routes of administration. Somewhat more research has been conducted with regards to the *in vivo* stability of CPPs. Several reports show that chemical alterations to the CPP sequence could improve its stability in plasma without altering the cell-penetrating capacity [109,110,111,112]. These modifications include N-methylation and the use of non-physiological D-amino acids which resulted in a decreased steric accessibility for proteolytic enzymes and the incompatibility of native enzymes to degrade D-amino acids, respectively [110,112]. As reported by Youngblood *et al*., a cheaper alternative to D-amino acids as the insertion of non-α-amino acids also increased their serum stability. They also observed that the instability of a CPP likely contributes to the endosomal trapping.

A different approach to CPPs is to target specific organelles, which could be beneficial if the cargo must be in a particular location to function. The Kelly group has developed CPPs that are also mitochondria-penetrating peptides (MPPs) [113]. This was achieved by the introduction of lipophilic residues and establishing a balance between the cationic character and hydrophobicity. This approach has already been successful in the study of cellular response to radical oxygen species [114]. However, MPPs compete with CPPs for cell entry and the absence of specific cellular delivery remains.

To conclude, as indicated by Howl *et al*., not every CPP is suited for every application [115]. There still is a major need to develop new quantitative assays and to compare the different existing methods in order to study and optimise both CPP-mediated delivery and endosomal escape efficiency *in vitro* and/or *in vivo*.
